# Are digital natives overconfident in their privacy literacy? Discrepancy between self-assessed and actual privacy literacy, and their impacts on privacy protection behavior

**DOI:** 10.3389/fpsyg.2023.1224168

**Published:** 2023-08-22

**Authors:** Shuai Ma, Chen Chen

**Affiliations:** ^1^School of International Business, Zhejiang Yuexiu University, Shaoxing, China; ^2^China Institute of Regulation Research, Zhejiang University of Finance & Economics, Hangzhou, China

**Keywords:** privacy literacy, digital natives, overconfidence, privacy protection behavior, privacy concern

## Abstract

Privacy literacy is recognized as a crucial skill for safeguarding personal privacy online. However, self-assessed privacy literacy often diverges from actual literacy, revealing the presence of cognitive biases. The protection motivation theory (PMT) is widely used to explain privacy protection behavior, positing that whether individuals take defensive measures depends on their cognitive evaluation of threats and coping capabilities. However, the role of cognitive biases in this process has been understudied in previous research. This study focuses on Chinese digital natives and examines the differential impacts of subjective and objective privacy literacy on privacy protection behavior, as well as the role of cognitive biases in privacy decision-making. The results show that there is no significant correlation between subjective and objective privacy literacy, and a bias exists. When privacy concern is used as a mediating variable, there are significant differences in the paths through which subjective and objective privacy literacy influence privacy protection behavior. Furthermore, privacy literacy overconfidence moderates the relationship between privacy concern and privacy protection behavior. The findings confirm the influence of cognitive biases in privacy behavior decision-making and extend the PMT. This study also calls for the government to enhance privacy literacy training for digital natives to improve their privacy protection capabilities.

## 1. Introduction

Today, issues such as ubiquitous surveillance and large-scale data collection (Greenwald, 2014), increasing commodification of information (Sevignani, 2016), and the blurring of public and private in the online environment (Masur, 2018) have made online privacy protection one of the most important topics in the digital economy era. However, many internet users are prone to leaking their personal data (Engels, 2019). For instance, in China, where the number of internet users has reached 1.051 billion as of June 2022, 21.8% of them have experienced personal information leaks (The 50th Statistical Report on China’s Internet Development, 2023), indicating a concerning situation. The internet is mainly used by young people, especially students, who tend to use internet resources for education, socializing, and entertainment. Young students are more exposed to information technology (IT) and the internet, as they have grown up in the digital era and are often referred to as digital natives. Digital natives are people who extensively use computers and the internet through various devices such as mobile devices, tablets, laptops, and desktop computers (Soroya et al., 2021). Although they are familiar with digital, they may not be proficient in technology, especially in terms of privacy and security (Till and Densmore, 2019). In addition, although digital natives are aware of various threats to privacy and security, few of them take specific measures to protect their privacy (Kurkovsky and Syta, 2010). In fact, Maier et al. (2023) found that digital natives are generally uncertain about the fate of their online data and feel powerless in controlling their own data. Instead, they rely more on government measures at the political and regulatory levels. As a result, strengthening the privacy literacy of digital natives has become a topic of common concern among scholars and governments.

Privacy literacy, defined as the ability to understand and manage privacy risks and security issues in the digital environment, has been recognized as a crucial factor influencing online privacy protection behavior (Park, 2013; Bartsch and Dienlin, 2016; Sindermann et al., 2021). It is considered a type of digital literacy, with higher levels of privacy literacy indicating greater proficiency in safeguarding personal privacy (Dinev and Hart, 2004). However, it has been found that users’ self-assessment of privacy literacy may not always align with their actual literacy level, leading to cognitive biases such as overconfidence or lack of confidence in privacy literacy (Brecht et al., 2012; Morrison, 2012). Notably, overconfidence, which is recognized as a prominent bias in human decision-making (de Bondt and Thaler, 1995), has been seldom explored in the context of privacy decision-making (Kokolakis, 2017). The Protection Motivation Theory (PMT) posits that individual protection motivation and behavior are influenced by threat appraisal and coping appraisal (Rogers, 1975; Floyd et al., 2000). This theory is also commonly used to explain privacy decision-making. For individuals with overconfidence in their privacy literacy, they often possess strong privacy protection self-efficacy, which facilitates the coping appraisal process. However, they may simultaneously underestimate the severity and vulnerability of perceived threats, which hinders the threat appraisal process. The overall impact of these cognitive biases on privacy protection behavior remains unclear. Therefore, investigating how cognitive biases affect privacy decision-making is a highly valuable research topic. Despite this, there is limited research that integrates both subjective and objective measures of privacy literacy in a comprehensive model to examine their differential impacts on privacy protection behavior. Moreover, although protection motivation theory (PMT) has been widely used to explain privacy decision-making, little attention has been given to the potential cognitive biases that may arise during users’ assessment of risk and coping abilities. Furthermore, most of the existing research has focused on internet users in developed countries, with limited in-depth investigation into the privacy literacy of digital natives in developing countries, where internet usage patterns and socio-cultural contexts may differ significantly from those in developed countries (Youn, 2009; Bal et al., 2015).

We are highly interested in investigating whether there is a discrepancy between the self-assessed (subjective) privacy literacy and the actual (objective) privacy literacy of digital natives, and whether this cognitive bias would impact their privacy decision-making behavior. Specifically, our research question is as follows:

*Q1:* Are digital natives overconfident in their privacy literacy? What is the relationship between subjective privacy literacy and objective privacy literacy?

*Q2:* Can both subjective privacy literacy and objective privacy literacy promote privacy protection behavior? What are the differences in their impact?

*Q3:* Does overconfidence affect privacy behavior decision-making?

To answer the above questions, we conducted a survey among digital natives in China, collected data, and conducted analysis. The remaining sections of this paper are structured as follows: the next section provides a literature review, and the theoretical assumptions and research model are also presented in this section; the third section presents the research design, including data collection and measurement of variables; followed by the analysis of research results; finally, the conclusion and discussion are presented, along with the limitations of this study and future research directions.

## 2. Literature review and theoretical hypotheses

### 2.1. Privacy literacy: subjective and objective

Initially, privacy literacy was commonly investigated as a cognitive ability by scholars. For instance, Langenderfer and Miyazaki (2009) defined privacy literacy as “users’ autonomous awareness of protecting their own privacy in online environments, as well as their understanding of the privacy risks associated with information interactions in such environments, primarily manifested as the ability to control internet privacy information.” Similarly, Givens (2016) conceptualized privacy literacy as users’ comprehension of how privacy information is tracked, used, collected, or lost in online environments, and its application in mitigating privacy challenges in email, social media, and web browsing. Subsequently, scholars began to define privacy literacy as the knowledge and skills that users possess to safeguard their privacy. For instance, Trepte et al. (2015) categorized privacy literacy into declarative knowledge and procedural knowledge. Declarative knowledge pertains to users’ cognitive understanding of privacy-related laws and regulations in online privacy protection, while procedural knowledge encompasses users’ practical know-how of privacy protection tools and settings, i.e., understanding how these tools can be utilized. Hence, privacy literacy can be conceptualized as users’ critical thinking abilities and capacity to engage in effective privacy protection behaviors (Bartsch and Dienlin, 2016), encompassing not only users’ cognitive understanding of online privacy protection, but also relevant skills and knowledge.

It is worth noting that Morrison (2012), in his study of privacy literacy among Canadian online social network users, categorized privacy literacy knowledge into self-assessed privacy literacy knowledge (SK) and objectively assessed privacy literacy knowledge (OK), and studied the differences between the two through comparison. Similarly, Brecht et al. (2012) also categorized privacy literacy into personally stated privacy literacy (PL-S) and actual privacy literacy (PL-A) in their research. Drawing on previous research, this study defines subjective privacy literacy (SPL) as “users’ self-assessed cognitive understanding and skills of online privacy protection,” and defines objective privacy literacy (OPL) as “users’ actual cognitive understanding and skills of online privacy protection.” When measuring subjective privacy literacy, scholars (Milne and Rohm, 2000; Brecht et al., 2012; Morrison, 2012; Bartsch and Dienlin, 2016) often use Likert scale items to measure, in which participants rate their own privacy literacy through self-assessment. When measuring objective privacy literacy, scholars (Brecht et al., 2012; Bartsch and Dienlin, 2016; Masur et al., 2017) often use objective knowledge items to measure, where a higher score indicates stronger objective privacy literacy. Masur et al. (2017) argued that subjective measurement of privacy literacy may be biased because people tend to overestimate or underestimate their own skills and knowledge, while objective knowledge scale is more objective. The discrepancy between subjective and objective privacy literacy can be used to measure participants’ overconfidence in their privacy literacy (Morrison, 2012). By applying the PMT theory for analysis, it can be observed that subjective privacy literacy represents an individual’s subjective cognition, reflecting their confidence and self-efficacy in privacy control. On the other hand, objective privacy literacy pertains more to an individual’s actual privacy skills and knowledge. Mastery of these skills and knowledge is essential for evaluating threat severity and vulnerability while also reducing response costs. However, the relationship between subjective privacy literacy and objective privacy literacy is complex and contingent upon the research context and situation, with varying findings reported in different studies (Brecht et al., 2012; Qiang and Xiao, 2021). Based on the above analysis, this paper proposes the following hypothesis:

*H1:* There is no significant correlation between subjective privacy literacy and objective privacy literacy.

### 2.2. Privacy literacy and privacy protection behavior

Privacy protection behavior can be defined as “computer-based actions taken by consumers to ensure information security” (Milne et al., 2009). Individuals can safeguard their online privacy by limiting information sharing and taking privacy protection measures (Büchi et al., 2016; Baruh et al., 2017). Privacy literacy has been identified as a significant predictor of privacy protection behavior, with higher levels of privacy literacy associated with enhanced privacy protection behavior. Subjective privacy literacy is often linked with the concept of privacy self-efficacy, as users who perceive their own privacy literacy as high tend to have greater confidence in their ability to protect their online privacy. Moreover, privacy self-efficacy has been found to significantly promote privacy protection behavior (LaRose et al., 2008; Milne et al., 2009). Additionally, research has confirmed that self-assessed privacy literacy positively influences privacy protection behavior (Bartsch and Dienlin, 2016). Therefore, we hypothesize that subjective privacy literacy has a positive effect on privacy protection behavior.

Regarding objective privacy literacy, studies have demonstrated that users with higher levels of privacy literacy knowledge are more likely to engage in information control compared to those with lower levels of knowledge (Park, 2013). In the context of social media, the importance of privacy literacy for data protection behavior has also been acknowledged, as users with higher privacy literacy are more likely to frequently change their privacy settings on Facebook (Bartsch and Dienlin, 2016). Other related studies have also confirmed that individuals with greater privacy literacy knowledge and skills are more likely to engage in privacy protection (Malandrino et al., 2013; Büchi et al., 2016; Ham and Nelson, 2016; Baruh et al., 2017; Ham, 2017). Therefore, this study proposes the following hypotheses:

*H2a:* Subjective privacy literacy is positively correlated with privacy protection behavior.

*H2b:* Objective privacy literacy is positively correlated with privacy protection behavior.

### 2.3. Mediating role of privacy concern

Privacy concern is recognized as a significant factor in explaining online privacy behavior (Choi et al., 2018), and therefore, this study investigates it as a potential mediating variable. As mentioned earlier, subjective privacy literacy is often linked with privacy self-efficacy, and previous research has found that self-efficacy enhances privacy concern (Mohamed and Ahmad, 2012; Lee et al., 2017), suggesting a positive correlation between subjective privacy literacy and privacy concern. In terms of objective privacy literacy, research has confirmed its positive effect on privacy concern. For instance, Baruh et al. (2017) found that users with higher privacy literacy levels exhibit higher levels of concern about privacy. Similarly, Prince et al. (2021) observed that users with higher privacy literacy displayed increased concerns and worries about privacy, possibly due to their heightened awareness of their ability to protect their online privacy as users, which makes them more vigilant about companies’ practices of collecting personal data and more aware of potential negative threats and consequences. Based on the above analysis, this study posits that users with higher privacy literacy, by virtue of their better understanding of institutions’ practices of collecting personal information online, are more likely to exhibit increased privacy concerns regarding potential risks to online privacy. Therefore, this study proposes the following hypotheses:

*H3a:* Subjective privacy literacy is positively correlated with privacy concern.

*H3b:* Objective privacy literacy is positively correlated with privacy concern.

Previous research has established the significant impact of privacy concerns on privacy protective behaviors. For instance, Youn’s (2009) study revealed that children aged 12–13 expressed high concerns about marketers’ data collection practices, which in turn influenced their engagement in privacy protective behaviors, such as providing false or incomplete information or browsing other websites. Feng and Xie’s (2014) research demonstrated that social media users adopted various privacy protective behaviors due to their heightened privacy concerns. Chen et al.’s (2017) study further confirmed that victims of online fraud tended to expand their privacy protective behaviors as a response to increased prediction of online privacy issues. Xie and Karan’s (2019) research found that privacy concerns about WeChat applications among college students had a significant and positive impact on their adoption of privacy protective behaviors. Similarly, Meng and Feng’s (2022) study confirmed that users of digital tourism platforms with higher privacy concerns were more likely to exhibit privacy protective behaviors. Based on the above analysis, it can be inferred that privacy literacy has the potential to enhance users’ privacy concerns, and these concerns, in turn, can further promote the adoption of privacy protective behaviors. Therefore, the following hypotheses are proposed:

*H4:* Privacy concerns are positively correlated with privacy protection behaviors.

*H5a:* Privacy concerns mediate the relationship between subjective privacy literacy and privacy protection behaviors.

*H5b:* Privacy concerns mediate the relationship between objective privacy literacy and privacy protection behaviors.

### 2.4. Moderating role of overconfidence

Overconfidence is one of the most prominent biases in human decision-making (de Bondt and Thaler, 1995). Overconfidence refers to the tendency of individuals to overestimate their perceived abilities in comparison to their actual abilities (Russo and Schoemaker, 1992), specifically in terms of overestimating their abilities, control, and chances of success (Moore and Healy, 2008). People may exhibit overconfidence in their perceived knowledge (McKenziea et al., 2008). This phenomenon can result in people showing indifference towards perceived risks (Ament and Jaeger, 2017), subsequently affecting their decision-making process. For example, entrepreneurs often demonstrate an excessive level of confidence in their skills, even when they acknowledge the potential risks of failure in the market (Busenitz, 1999). The study conducted by Morrison (2012) demonstrated that 47.5% of the participants overestimated their privacy knowledge, providing empirical evidence for the existence of overconfidence in the realm of privacy decision-making. Overconfidence primarily emerges in situations characterized by unknown probabilities (Russo and Schoemaker, 1992), and privacy decision-making is subject to various uncertain factors (Acquisti and Grossklags, 2005). Some scholars have investigated the influence of overconfidence on privacy decision-making behavior. For instance, Wagner and Mesbah (2019) found that overconfidence mitigated the negative relationship between perceived risk and intention to use smartphone applications. Thus, individuals who are overconfident in the context of privacy decision-making may underestimate or disregard privacy risks they face (Wagner and Mesbah, 2019), overestimate their privacy controls (Gino et al., 2011; Chen and Koufaris, 2015), and engage in more information disclosure or fewer privacy protection behaviors. Based on these observations, the following hypotheses are proposed in this study:

*H6a(b):* Overconfidence negatively moderates the relationship between subjective (objective) privacy literacy and privacy concern.

*H7a(b):* Overconfidence negatively moderates the relationship between subjective (objective) privacy literacy and privacy protection behavior.

*H8:* Overconfidence negatively moderates the relationship between privacy concern and privacy protection behavior.

**Figure 1:**
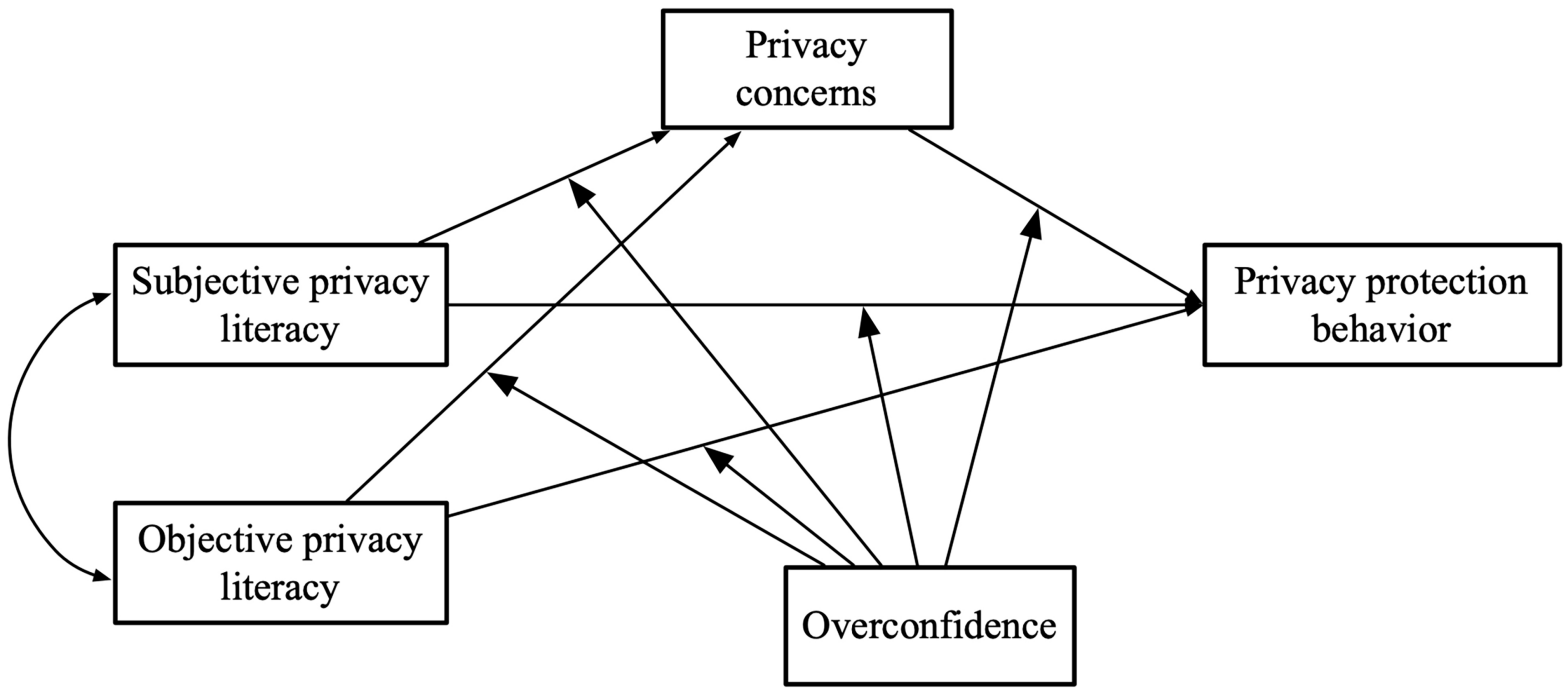
Theoretical model.

Based on the above analysis, the research framework of this study is shown in Figure 1.

## 3. Research design

### 3.1. Data collection and implementation

The study’s target population comprises digital natives, who are individuals that have grown up in the digital world and are proficient in using information and communication technologies (Palfrey et al., 2009). In China, the “post-2000 generation” represents a true digital native population, as they have been born into a world surrounded by digital technologies. Given that undergraduate students are frequent internet users, the survey was conducted at a university in eastern China, following the approach of Soroya et al. (2021), where students are typical representatives of the “post-2000 generation.” We employed a stratified random sampling method to randomly select participants from various academic majors and grades. A total of 462 questionnaires were collected and subsequently subjected to meticulous screening. Questionnaires that did not meet the criteria were excluded, such as those with response times shorter than 2 min (as very brief response times may compromise the questionnaire’s reliability) and those displaying clear patterns in the answers (e.g., selecting “uncertain” for all items in a scale). The final sample included 401 participants with valid responses. Descriptive statistics and correlation analysis were conducted using SPSS, and structural equation modeling analysis was performed using AMOS.

### 3.2. Measurement of variables

#### 3.2.1. Subjective privacy literacy (SPL)

The SPL scale was adapted from three items by Morrison (2012) to reflect respondents’ subjective evaluation of their own privacy literacy. A 5-point Likert scale was used, and respondents rated their perceived subjective privacy literacy on the following items: “Compared to most people you know, how do you rate your knowledge of how organizations collect and manage your personal information?” (1 = one of the least knowledgeable, 5 = one of the most knowledgeable); “Overall, I have a very good understanding of how organizations collect and manage my personal information.” (1 = strongly disagree, 5 = strongly agree); “I have a very good understanding of how companies collect and manage the information I provide in online social networks.” (1 = strongly disagree, 5 = strongly agree). To minimize bias, the SPL items were tested before the OPL items. The scale was found to have internal consistency, as the value of Cronbach’s α (α = 0.825) exceeded the acceptable minimum of 0.7 (Nunnally, 1978).

#### 3.2.2. Objective privacy literacy (OPL)

The OPL scale consisted of 10 True/False/Do not know items (Appendix A), adapted from Morrison (2012) and Masur et al. (2017), and modified based on Chinese laws and regulations. The total score of correctly answered OPL items was used as the value of OPL (ranging from 0 to 10). Given the way this measure was constructed, internal consistency estimation is not reported in this study.

#### 3.2.3. Privacy concerns (PC)

The PC scale was adapted from Chen (2018) and consisted of three items, rated on a 5-point Likert scale, including “I am concerned that the information submitted to websites or apps may be misused,” “I am concerned about providing personal information to websites or apps because others may see and use this information,” and “I am concerned about providing personal information to websites or apps because this information may be used in unforeseen ways.” Participants were asked to rate the extent to which they agreed with these statements in relation to their own situation, ranging from 1 (strongly disagree) to 5 (strongly agree). The scale was found to have internal consistency, as the value of Cronbach’s α (α = 0.933) exceeded the acceptable minimum of 0.7 (Nunnally, 1978).

#### 3.2.4. Privacy protection behavior (PPB)

Drawing on the approach of Kezer et al. (2016) and Sindermann et al. (2021), participants are required to indicate whether they have engaged in specific types of privacy protection behaviors from a list of 10 items (Appendix B). The total number of privacy protection behaviors endorsed by participants is used to compute the value of PPB. Due to the nature of how this measurement is derived, internal consistency estimates are not reported in this study.

## 4. Results

### 4.1. Descriptive analysis

**Figure 2:**
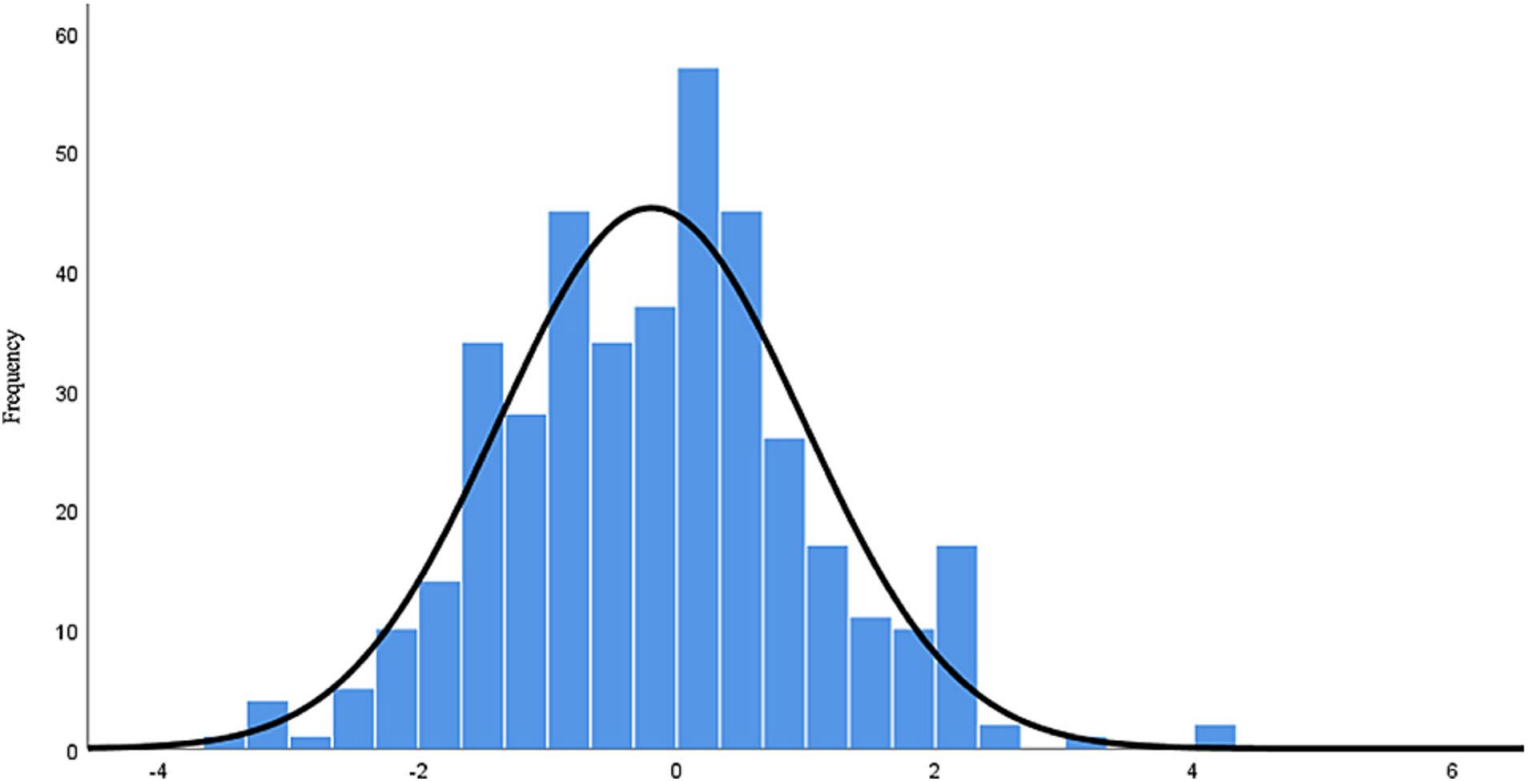
Calibration frequency distribution.

Descriptive statistics of SPL indicate that respondents perceive their knowledge of online privacy to be neutral (M = 2.96, SD = 0.76). Among the respondents, 35.9% have a lower SPL score (SPL score less than 3), 30.4% have a higher SPL score (SPL score greater than 3), and 33.7% have an SPL score of 3. These results suggest that the SPL is not high for the sample. Descriptive statistics of OPL show that the average score is M = 5.39 (SD = 2.304), with 5.24% of respondents (*N* = 21) scoring 0 on OPL, indicating that they did not answer any privacy knowledge questions correctly. Only 0.996% (*N* = 4) obtained a score of 10, indicating perfect accuracy in privacy knowledge. 18.70% of respondents (*N* = 75) scored 5, accurately answering half of the questions. To answer Q1, following the approach of Morrison (2012), calibration was performed by calculating the difference between SPL and OPL. As the basic measurements used different scales, conversion of OPL and SPL values was necessary for comparison. The lowest SPL score that reflects a complete lack of subjective privacy literacy is 1, while the lowest OPL score is 0. Therefore, the calibration measurement was calculated by first recoding the SPL scores to a range of 0–4, and then converting the OPL scores to a range of 0–4 (OPL score/10*4). Finally, the calibrated value was obtained by subtracting the converted OPL scores from the converted SPL scores. If this value is greater than 0, it reflects overconfidence in privacy literacy, whereas if it is less than 0, it indicates a lack of confidence in privacy literacy. Descriptive statistics show that the calibration ranged from −3.6 to 4, with a mean of M = −0.196 (SD = 1.18). The frequency distribution of calibration shows that 92.8% (*N* = 372) of respondents calibrated their privacy literacy incorrectly. Among them, 53.1% (*N* = 213) had calibration scores below zero, 39.7% (*N* = 159) had calibration scores above zero, and 7.2% (*N* = 29) had a complete calibration of privacy literacy. These results indicate that errors in privacy literacy calibration are evident. Overall, respondents tend to lack confidence in their privacy literacy, but the situation is not dire. Figure 2 shows the histogram of the calibration variable, which generally conforms to a normal distribution.

The descriptive statistics for the main variables reveal that respondents exhibit a relatively high level of privacy concern (M = 3.82, SD = 0.801), indicating a general sense of worry about privacy issues. Privacy protection behavior (M = 6.18, SD = 2.875) shows a moderate level, but compared to privacy concern, it exhibits higher variability, suggesting significant differences in privacy protection behavior among respondents.

### 4.2. Structural model analysis

Based on the calculations using AMOS, the fit indices of the structural model in this study are as follows: (CMIN/DF) = 1.084, GFI = 0.989, NFI = 0.989, IFI = 0.999, CFI = 0.999, RMSEA = 0.014. All the indices are within reasonable range, as they are much higher than the traditional threshold values suggested by Fornell and Larcker (1981). This confirms the acceptability of the research model’s feasibility.

### 4.3. Path analysis and hypothesis testing

**Figure 3:**
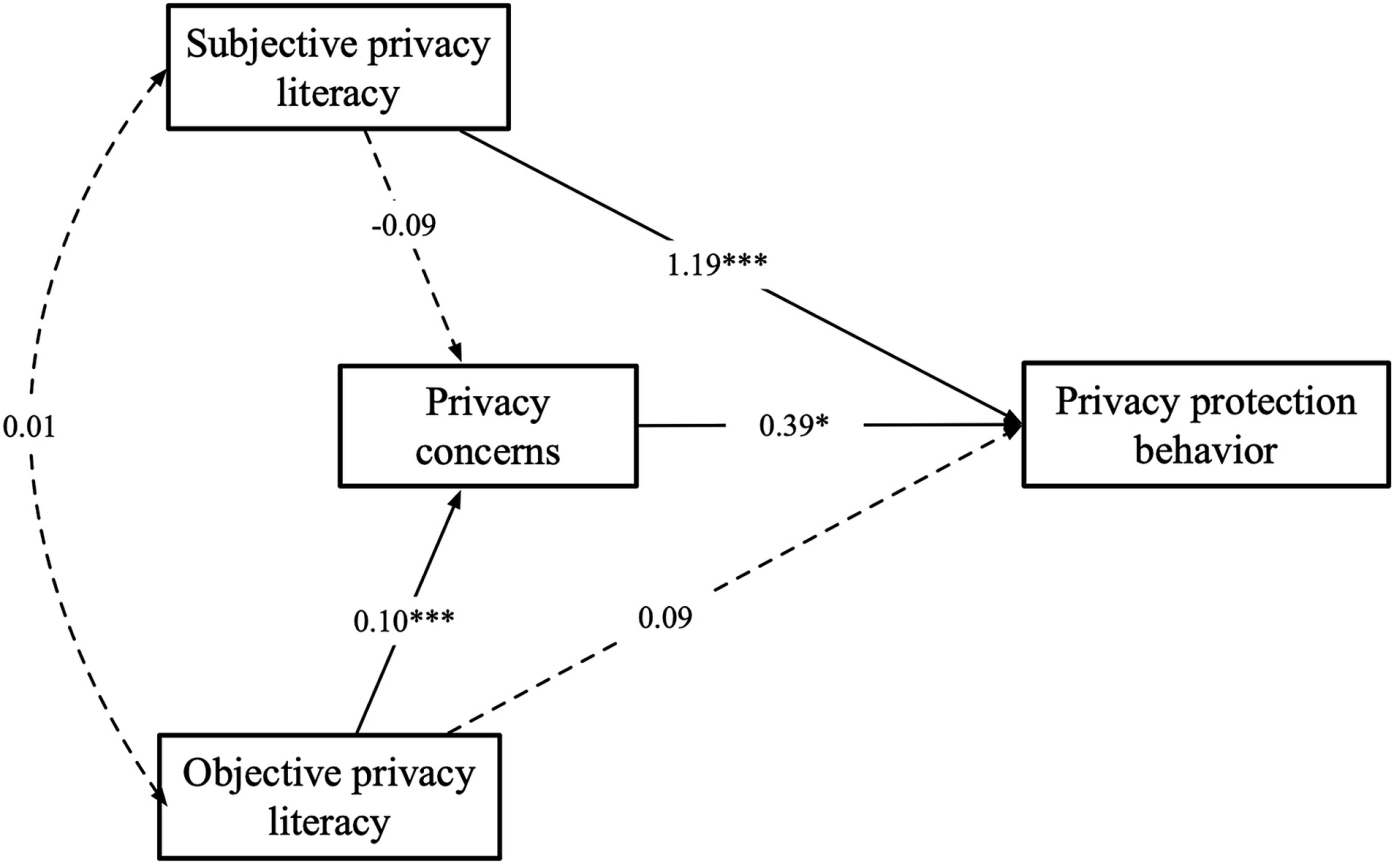
The final model of path analysis. *p < 0.05, **p < 0.01, ***p < 0.001.

The results of the path analysis of the SEM model constructed based on the theoretical framework were used to test the hypotheses. The results from Figure 3 and Table 1 show that there is no significant correlation between subjective privacy literacy and objective privacy literacy (*H1*: B = 0.009, SE = 0.071, *p* > 0.05), indicating that there is no relationship between subjective privacy literacy and objective privacy literacy, thus supporting hypothesis *H1*. There is no significant correlation between subjective privacy literacy and privacy concerns (*H3a*: B = −0.089, SE = 0.074, *p* > 0.05), indicating that hypothesis *H3a* is not supported. There is a positive correlation between objective privacy literacy and privacy concerns (*H3b*: B = 0.099, SE = 0.017, *p* < 0.001), indicating that objective privacy literacy has a positive impact on privacy concerns, thus supporting hypothesis *H3b*. Privacy concerns are positively correlated with privacy protective behaviors (*H4*: B = 0.393, SE = 0.184, *p* < 0.05), indicating that privacy concerns can predict privacy protective behaviors, thus supporting hypothesis *H4*. Subjective privacy literacy is positively correlated with privacy protective behaviors (*H2a*: B = 1.185, SE = 0.268, *p* < 0.001), indicating that subjective privacy literacy can directly influence privacy protective behaviors, thus supporting hypothesis *H2a*. However, there is no significant correlation between objective privacy literacy and privacy protective behaviors (*H2b*: B = 0.095, SE = 0.063, *p* > 0.05), indicating that hypothesis *H2b* is not supported. This suggests that objective privacy literacy may not directly influence privacy protective behaviors when privacy concerns are considered, and there may be an intermediate path that needs further analysis.

**Table 1:** Results of the path analysis (*N* = 401).

Path	Standardized coeff.	Unstandardized coeff.	S.E.	C.R.	*p*
SPL → PC	−0.063	−0.089	0.074	−1.203	0.229
OPL → PC	0.282	0.099	0.017	5.720	***
PC → PPB	0.111	0.393	0.184	2.136	*
SPL → PPB	0.238	1.185	0.268	4.420	***
OPL → PPB	0.076	0.095	0.063	1.508	0.132

*p < 0.05, **p < 0.01, ***p < 0.001.

### 4.4. Mediation effect analysis

Bootstrap method was used in this study to test the mediation effect of privacy concerns. As bias-corrected percentile bootstrap method is superior to percentile bootstrap method (Pituch and Stapleton, 2008), only the results of bias-corrected percentile bootstrap are reported in Table 2. The total effect and direct effect of the first mediation path do not include 0 in the 95% CI, but the indirect effect includes 0, indicating that the total effect and direct effect are significant, but the indirect effect is not significant. This suggests that subjective privacy literacy directly influences privacy protective behaviors, but privacy concerns do not mediate this relationship, thus not supporting hypothesis *H5a*. In contrast, the total effect and indirect effect of the second mediation path do not include 0 in the 95% CI, but the direct effect includes 0, indicating that the total effect and indirect effect are significant, but the direct effect is not significant. This suggests that privacy concerns mediate the relationship between objective privacy literacy and privacy protective behaviors, thus supporting hypothesis *H5b*.

**Table 2:** Mediating effect test (*N* = 401, Bootstrap samples 5,000).

Mediating path	Type of effect	Coeff.	S.E.	Bias − corrected 95% CI	
				Lower	Upper
SPL → PC → PPB	Total effect	1.150	0.273	0.615	1.703
	Indirect effect	−0.035	0.048	−0.166	0.034
	Direct effect	1.185	0.280	0.636	1.756
OPL → PC → PPB	Total effect	0.134	0.058	0.020	0.246
	Indirect effect	0.039	0.020	0.007	0.085
	Direct effect	0.095	0.061	−0.026	0.213

### 4.5. Moderation effect analysis

Based on the calibration variable of privacy literacy, samples with a calibration value of privacy literacy greater than 0 were defined as the “overconfidence” group, and samples with a corresponding calibration variable value less than 0 were defined as the “non-overconfidence” group. Then, multi-group analysis was conducted using Amos. The results showed that the unconstrained model and the constrained model were significantly different ($\chi^{2}=18.932$, *p* < 0.05), indicating that overconfidence indeed played a moderating role. Furthermore, Table 3 reports the path coefficients and significance of differences between the overconfidence and non-overconfidence groups. The results showed that although there were differences in path coefficients between the two groups for all paths, only the path coefficients between privacy concern and privacy protection behavior, and between objective privacy literacy and privacy protection behavior were statistically significantly different. However, since the relationship between objective privacy literacy and privacy protection behavior was not significant in both the overconfidence and non-overconfidence groups, this indicates that overconfidence only moderates the relationship between privacy concern and privacy protection behavior. Therefore, *H6a(b)* and *H7a(b)* are not supported, while *H8* is supported.

**Table 3:** Multigroup analysis.

Path		Over confidence			Non-overconfidence		*Z*-score
	Coeff.	C.R.	*p*-Values	Coeff.	C.R.	*p*-Values	
SPL → PC	0.076	0.527	0.598	−0.030	−0.285	0.776	−0.594
OPL → PC	0.022	0.624	0.532	0.067	2.062	*	0.938
PC → PPB	−0.345	−0.987	0.324	1.061	3.708	***	3.112**
SPL → PPB	1.087	1.755	0.079	1.595	3.648	***	0.671
OPL → PPB	0.232	1.562	0.118	−0.218	−1.729	0.084	−2.309*

*p < 0.05, **p < 0.01, ***p < 0.001.

## 5. Conclusion and discussion

### 5.1. Discussion

Are digital natives overconfident in their privacy literacy? This research question was examined using calibrated measurement variables. The results indicated that 39.7% of respondents had calibration scores greater than 0, suggesting a significant portion of individuals demonstrated overconfidence in their privacy literacy. However, it is important to note that this proportion did not exceed 50%. Interestingly, 53.1% of respondents had calibration scores lower than 0, indicating that over half of the respondents underestimated (i.e., lacked confidence in) their privacy literacy. Notably, this proportion was higher than the value reported in Morrison’s (2012) study. One possible explanation for this phenomenon is that the increasing severity of privacy leaks and breaches in China has eroded the confidence of digital natives in their ability to effectively control their privacy information online. Furthermore, our study demonstrates a lack of significant correlation between subjective privacy literacy and objective privacy literacy, which aligns with the findings reported by Qiang and Xiao (2021). This implies that individuals who claim to have a good grasp of privacy control may not actually possess the level of knowledge and skills they assert, suggesting a potential overestimation of their abilities.

The findings of this study highlight that objective privacy literacy has a positive influence on privacy concerns, which differs from the results reported by Youn (2009), but aligns with the findings of Baruh et al. (2017) and Prince et al. (2021). According to the PMT theory (Rogers, 1975; Floyd et al., 2000), the severity and vulnerability of perceived threat can lead to worry and anxiety, thereby promoting protection motivation. Users with higher levels of objective privacy literacy possess a better understanding of how institutions collect and use user information, enabling them to be more attentive to the risks and threats to online privacy, thus showing greater concern for online privacy issues. However, research results indicate no significant correlation between subjective privacy literacy and privacy concern, neither positive nor negative (Brecht et al., 2012). As previously discussed, subjective privacy literacy primarily reflects self-efficacy and has more influence on the coping appraisal process rather than the threat appraisal process, which may explain its lack of direct association with privacy concern. Moreover, the results of this study indicate that privacy concerns positively predict privacy protection behaviors, corroborating the findings of Youn (2009), Feng and Xie (2014), Chen et al. (2017), Xie and Karan (2019), and Meng and Feng (2022), suggesting that users who express higher levels of privacy concerns are more inclined to engage in privacy protection behaviors.

Is there a distinction in the impact of subjective privacy literacy and objective privacy literacy on privacy protection behavior? The results of this research indicate that subjective privacy literacy can directly impact privacy protection behavior, but it does not exert an indirect impact through privacy concerns. On the other hand, when privacy concerns are taken into consideration, objective privacy literacy does not directly impact privacy protection behavior, but instead exerts influence through privacy concerns. One possible explanation is that individuals with higher subjective privacy literacy tend to have greater privacy self-efficacy, which directly influences privacy protection motivation and behavior. However, subjective privacy literacy may have little or no involvement in threat appraisal, and therefore, it does not directly trigger privacy concern. In contrast, objective privacy literacy exerts its impact on protection motivation and behavior through threat appraisal, and in this study, it demonstrated a full mediating effect on privacy concerns. This result can also be elucidated by the “cognition-attitude-behavior” model from cognitive psychology, which suggests that individuals’ attitudes towards a specific action depend on their cognition of the causes and effects, and attitudes strongly predict behavioral intentions (Murchison, 1930). Subjective privacy literacy encompasses both understanding and cognition of privacy, as well as attitudes towards privacy management, thus it can directly influence privacy protection behavior. However, for objective privacy literacy, it represents mere understanding of privacy knowledge and skills, and this cognition needs to be translated into attitude (such as privacy concern) in order to generate corresponding privacy protection behavior.

Does overconfidence impact behavioral decision-making? The findings of this study reveal that overconfidence acts as a negative moderator in the relationship between privacy concerns and privacy protection behaviors. As discussed at the beginning of the article, how does the cognitive bias of overconfidence affect privacy decision-making? According to the PMT theory (Rogers, 1975; Floyd et al., 2000), privacy decision-making is influenced by two processes: threat appraisal and coping appraisal. If an individual is overconfident about their privacy literacy, they are likely to have strong self-efficacy, thereby increasing the likelihood of engaging in privacy protection behaviors. However, this cognitive bias can also lead the individual to underestimate the severity and vulnerability of perceived threats, thereby reducing the likelihood of engaging in privacy protection behaviors. Our research findings seem to suggest that overconfidence plays a predominantly “negative role.” When individuals exhibit overconfidence, the impact of privacy concern on privacy protection behavior weakens. As hypothesized, individuals with overconfidence face challenges in effectively translating their privacy concerns into privacy protection behaviors due to an overestimation of their control over privacy. These results further corroborate the significance of cognitive biases in the domain of privacy decision-making.

Privacy literacy, being a malleable skill (Goldie, 2006), can be improved through targeted education and training interventions. Recent research by Desimpelaere et al. (2020) has confirmed that training can significantly enhance children’s understanding of organizational data practices, empowering them to better protect their personal privacy. Building on these findings, we emphasize the critical importance of strengthening the privacy knowledge and skills of digital natives in today’s digital age. Firstly, providing digital natives with relevant training on organizational practices can help them grasp how organizations and data service providers collect and utilize their data for business purposes, enabling a comprehensive understanding of the root causes of privacy issues. Secondly, enhancing training on privacy protection skills, such as browser privacy settings, can enhance digital natives’ ability to effectively safeguard their online privacy. Lastly, strengthening education on privacy protection laws and regulations for digital natives can guide them to legally protect their online privacy through proper complaint and litigation procedures, rather than relying solely on avoidance or tolerance strategies. Meanwhile, in order to fundamentally eliminate institutions’ infringement on citizens’ privacy, it is necessary for the government to strengthen legislative efforts in the field of privacy protection. Currently, government legislation lags behind the rapidly evolving forms and types of privacy infringements in the information society, requiring urgent legislative actions and legal revisions to address various loopholes in the protection of citizens’ online privacy and effectively safeguard the online surfing security of internet users.

There are several limitations to this study that warrant consideration. Firstly, the sample of this study was limited to undergraduate students from universities, who may not fully represent all digital natives in China, despite being an active and representative group. Future research could adopt a nationwide survey approach and consider factors such as education level in the analysis to enhance the generalizability of the findings. Secondly, although privacy concerns were selected as the mediator variable in this study, it is important to note that privacy literacy may impact privacy protection behaviors through other pathways as well. Future studies could explore alternative variables and pathways to further elucidate the complex relationship between privacy literacy and privacy behavior.

Despite these limitations, this study has made significant academic contributions. Notably, it incorporated both subjective and objective privacy literacy into the research model, shedding light on their differential impacts on privacy behavior. Moreover, this study extended the protection motivation theory (PMT) by investigating the influence of cognitive biases, such as overconfidence, on privacy decision-making, thereby enriching the existing literature on privacy behavior.

### 5.2. Conclusion

The issue of online privacy protection has long been a prominent topic of research and policy focus for scholars and governments worldwide. This study aimed to investigate the differential effects of subjective and objective privacy literacy among Chinese digital natives on privacy protection behavior, as well as the influence of overconfidence in privacy decision-making. The study findings revealed that there was no significant correlation between subjective and objective privacy literacy among Chinese digital natives, with a notable bias between the two. A substantial proportion of participants exhibited overconfidence in their privacy literacy. Furthermore, subjective privacy literacy was not significantly correlated with privacy concern, but positively correlated with privacy protection behavior. On the other hand, objective privacy literacy showed a positive correlation with privacy concern, and privacy concern acted as a mediator between objective privacy literacy and privacy protection behavior. Additionally, the relationship between privacy concern and privacy protection behavior was found to be moderated by overconfidence. Overall, the results of this study contribute to the existing literature on the complex relationship between privacy literacy and privacy protection behavior, and shed light on the influence of cognitive distortions and biases in privacy decision-making.

## Data availability statement

The raw data supporting the conclusions of this article will be made available by the authors, without undue reservation.

## Ethics statement

Ethical review and approval was not required for the study on human participants in accordance with the local legislation and institutional requirements. Written informed consent from the patients/participants or patients/participants' legal guardian/next of kin was not required to participate in this study in accordance with the national legislation and the institutional requirements.

## Author contributions

SM is responsible for the overall research design, thesis writing, collation of the questionnaire, and data analysis. CC is responsible for the proofreading and article touch-up. All authors contributed to the article and approved the submitted version.

## Funding

This research was funded by Zhejiang Provincial Philosophy and Social Science Planning Project, grant number 22NDQN282YB.

## Conflict of interest

The authors declare that the research was conducted in the absence of any commercial or financial relationships that could be construed as a potential conflict of interest.

## Publisher’s note

All claims expressed in this article are solely those of the authors and do not necessarily represent those of their affiliated organizations, or those of the publisher, the editors and the reviewers. Any product that may be evaluated in this article, or claim that may be made by its manufacturer, is not guaranteed or endorsed by the publisher.
