# Supplementary material for: Are digital natives overconfident in their privacy literacy? Discrepancy between self-assessed and actual privacy literacy, and their impacts on privacy protection behavior
Source: Front Psychol. 2023 Aug 22;14:1224168. doi: 10.3389/fpsyg.2023.1224168 (PMC10477717; doi:10.3389/fpsyg.2023.1224168)
Supplement: Supplementary file 1 [file Data_Sheet_1.pdf]

Appendix A—Objective Privacy Literacy (OPL) Scores by Question

Below there are a few statements about Internet and online privacy. For each statement, please indicate whether you think it is true or false.

1=True

2=False

Q1	Organizations create user profiles by integrating user data collected from different websites.
Q2	Organizations store collected user information properly, with no risk of user privacy leakage.
Q3	Apps or websites usually specify in their privacy policies the personal information they need to collect.
Q4	Organizations do not transfer or sell collected user information to other institutions.
Q5	Organizations do not excessively collect users' personal information without authorization.
Q6	When personal information is unlawfully collected and used, individuals can report and lodge complaints with relevant authorities.
Q7	Individuals have the right to inquire about and copy their personal information from organizations.
Q8	In certain situations, organizations may provide your information to law enforcement personnel without your consent.
Q9	Organizations may change the purposes, methods, and scope of personal information processing without notifying users.
Q10	If users do not agree to the collection of non-essential personal information, organizations may refuse to provide the basic business functions of the product.

Appendix B—Privacy Protection Behaviors Taken online

Have you ever done the following because you are concerned about your privacy online?

1=Yes

2=No

1. Submitting false information online.

2. Providing incomplete personal information on the internet.

3. Using online privacy protection software.

4. Browsing the internet using the privacy mode of a browser.

5. Setting more secure and less susceptible account passwords.

6. Clearing internet browsing history.

7. Deleting cookies.

8. When websites or apps request personal information, leave the site directly.

9. When websites or apps request certain information or authorize specific functions, refuse to provide.

10. During the information filling process, stopping due to fear of privacy leakage.
